# Comparison of transmission of *Papaya leaf curl China virus* among four cryptic species of the whitefly *Bemisia tabaci* complex

**DOI:** 10.1038/srep15432

**Published:** 2015-10-21

**Authors:** Tao Guo, Qi Guo, Xi-Yun Cui, Yin-Quan Liu, Jian Hu, Shu-Sheng Liu

**Affiliations:** 1 Ministry of Agriculture Key Laboratory of Agricultural Entomology, Institute of Insect Sciences, Zhejiang University, Hangzhou, 310058, China; 2 Yunnan Provincial Key Lab of Agricultural Biotechnology, Biotechnology and Germplasm Resources Institute, Yunnan Academy of Agricultural Sciences, Kunming, 650223, China

## Abstract

Begomoviruses are transmitted by cryptic species of the whitefly *Bemisia tabaci* complex, often in a species-specific manner. *Papaya leaf curl China virus* (PaLCuCNV) has been recorded to infect several crops including papaya, tomato and tobacco in China. To help assess the risks of spread of this virus, we compared the acquisition, retention and transmission of PaLCuCNV among four species of whiteflies, Middle East-Asia Minor 1 (MEAM1), Mediterranean (MED), Asia 1 and Asia II 7. All four species of whiteflies are able to acquire, retain and transmit the virus, but with different levels of efficiency. Transmission tests using tomato as the host plant showed that MEAM1 transmitted PaLCuCNV with substantially higher efficiency than did MED, Asia 1 and Asia II 7. Furthermore, accumulation of PaLCuCNV in the whiteflies was positively associated with its efficiency of transmitting the virus. Altogether, these findings indicate that MEAM1 is the most efficient vector for PaLCuCNV in the four species of whiteflies, and suggest that risks of PaLCuCNV pandemics are high in regions where MEAM1 occurs.

The whitefly *Bemisia tabaci* (Gennadius) (Hemiptera: Aleyrodidae) is a species complex of over 35 cryptic species, recently ascertained by evidence provided by molecular phylogenetic analyses and reciprocal crossing experiments between genetic groups^1^,^2^,^3^. Whiteflies of this species complex cause damage to plants through direct feeding and transmission of plant viruses^4^,^5^,^6^. Some species of this complex, such as the two invasive species Middle East–Asia Minor 1 (MEAM1) and Mediterranean (MED), formerly often referred to as the B and Q “biotypes”, have caused extensive damage to world agriculture due to their rapid and widespead invasions around the globe in the past 30 years^1^,^5^.

Most of the whitefly-transmitted viruses belong to the genus *Begomovirus* (family *Geminiviridae*), which are one of the most important groups of plant viruses in tropical and subtropical regions of the world^6^. Members of this virus family have a circular single-stranded DNA genome packaged within twinned icosahedral particles, 20–30 nm in size. The genus *Begomovirus*, with its more than 200 species, is exclusively transmitted by whiteflies of the *B. tabaci* complex in a persistent-circulative mode^6^,^7^,^8^. In China, over 40 species of begomoviruses have been recorded in the past 50 years and some of them have caused severe losses to a range of crops including among others tomato, tobacco, and papaya, particularly in the past 15 years following the invasions of MEAM1 and MED^9^,^10^,^11^,^12^. The begomovirus *Papaya leaf curl China virus* (PaLCuCNV) was a newly isolated monopartite begomovirus without satellite DNA in Guangxi Province of China, and has been causing a severe leaf curl disease on papaya in southern China^13^. In recent years, PaLCuCNV has spread to tomato in Guangxi, Fujian (GenBank accession no.: AM691554), Henan, Yunnan and Sichuan Province of China^12^,^14^,^15^.

In China, so far 16 species of the *B. tabaci* complex have been recorded, consisting of 14 indigenous species and the two invasive species MEAM1 and MED^16^,^17^. Most of the *B. tabaci* fauna occurs in the southern, southeastern and southwestern regions of China^16^. The diversification and spread of begomoviruses are supposedly related to the genetic and phenotypic variability of *B. tabaci*^18^. Studies have shown that different species of the *B. tabaci* complex may transmit a given begomovirus with various levels of efficiency or specificity, and each of the whitefly species may only be able to transmit certain viruses^19^,^20^,^21^,^22^,^23^.

While PaLCuCNV has been spreading in China, no data are yet available on the efficacy of putative vectors of PaLCuCNV, and how the virus got disseminated^12^,^13^. To study the role of whitefly vectors in the epidemiology of PaLCuCNV, we conducted laboratory experiments to compare the acquisition, retention and transmission of PaLCuCNV by species of the *B. tabaci* complex. We chose for the tests four species of whiteflies, two invasive MEAM1 and MED because they are the most abundant whiteflies across China including Guangxi and in many other countries, and two indigenous Asia 1 and Asia II 7 because they are among the most abundant of the indigenous whiteflies in south and southwestern regions of China, where PaLCuCNV was firstly recorded^12^,^13^,^16^,^17^. Our data indicate that among the four species of whiteflies MEAM1 is the most efficient vector of PaLCuCNV, and this finding has important implications in understanding and predicting the epidemiology of this virus in the field.

## Materials and Methods

### Whitefly cultures and virus clone

Four cultures of four cryptic species of the whitefly *B. tabaci* complex were originally collected from four localities in China and were maintained in the laboratory (Table 1). All cultures were reared on cotton (*Gossypium hirsutum* cv. Zhe-Mian 1793) as cotton is a non-host plant of PaLCuCNV and a suitable host for all the four whitefly species. The insects were reared in a climate-controlled chamber at 26 ± 1 °C, LD 14:10 h and 60 ± 10% relative humidity. Half of the cotton plants in a culture were replaced by fresh ones every 60 days in an alternate manner so as to offer the whiteflies some new plants to colonize every 30 days. The purity of each of the four cultures was examined every three to five generations by randomly sampling 30–50 adults as described in Qin *et al.* (2013)^24^ by using the mitochondrial cytochrome oxidase I (*mtCOI* ) PCR-RFLP technique and further confirmed by sequencing of 5–10 individuals. To obtain virus-infected tomato plants (*Solanum lycopersicum* Mill. cv. Hezuo 903), an infectious clone of PaLCuCNV was used to generate sources of inoculum^12^ and plants at the 3–4 true-leaf stage were agro-inoculated with PaLCuCNV as described previously^25^. Plants were cultivated to the 6–7 true leaf stage for further experiments. Infection of tomato plants was assessed by appearance of typical disease symptoms and presence of viral DNA. All plants were grown in a greenhouse at controlled temperatures of 20–30 °C, 14 L: 10 D (natural lighting supplemented with artificial light), 50–70% relative humidity. Prior to a test, all leaves from each plant were checked with a 20× hand lens to ensure that plants were insect-free.

### Acquisition of PaLCuCNV by whiteflies

For each of the four whitefly species, approximately 300 newly emerged (0–24 h), non-viruliferous adult whiteflies were drawn from their respective culture and transferred to feed on two PaLCuCNV-infected tomato plants enclosed in an insect-proof cage. The lower two older leaves of the plants, which showed mild or no symptoms, were covered by an insect-proof mesh bag one day before the whitefly transfer. Care was taken to gently place the adults evenly onto all the exposed leaves. Following the adult transfer, we randomly collected 10 adults from leaves of the two plants at the end of 13 designated acquisition access periods (AAPs; 0, 0.5, 1, 1.5, 2, 3, 4, 6, 8, 10, 12, 24, and 48 h) on the plants. The adults collected were stored at −20 °C and later assayed individually for detectable PaLCuCNV DNA by conventional PCR (hereafter PCR).

### Retention of PaLCuCNV by whiteflies

For each of the four whitefly species, approximately 400 newly emerged (0–24 h) non-viruliferous adults were transferred to feed on two PaLCuCNV-infected tomato plants enclosed in an insect-proof cage for 48 h AAP to obtain a 100% viruliferous whitefly population as determined above. Then viruliferous adults of each species were collected and released to feed on a healthy cotton plant, a non-host of PaLCuCNV. Following the initial release, a group of 10 live adults were collected at 0, 0.5, 1, 2, 3, 4, 5, 10, 15, 20, 25, 30 and 40 d respectively. During the collection process, the adults remaining on the cotton were transferred onto a new cotton plant at 15 and 30 d respectively to avoid emergence of new adults. To examine whether the virus might survive in the cotton leaves and whiteflies might acquire the virus through feeding on cotton, we also made detection of the virus in the cotton leaves that had been fed by viruliferous whiteflies for 1, 5, and 10 d. The insects and leaf samples collected were stored at −20 °C and later assayed individually by PCR for detection of PaLCuCNV DNA.

### Transmission of PaLCuCNV to tomato plants by whiteflies

For each of the four whitefly species, approximately 500 newly emerged (0–24 h) adults drawn from their respective cultures were released to feed on two PaLCuCNV-infected tomato plants for 48 h. The viruliferous adults were then collected and inoculated in groups of 1, 5 or 10 to feed on the second leaf from the bottom of an uninfected tomato plant at the three true-leaf stage using a clip-cage, for a 48 h inoculation access period. To quantify PaLCuCNV in these whiteflies after a 48 h AAP plus a 48 h inoculation access period, we randomly collected 15 female adults from each of the treatments with 10 adults per plant and conducted quantitative PCR (qPCR) for each individual. The plants were then sprayed with imidacloprid at a concentration of 50 mg/l to kill all the adults and eggs and kept for symptom development in insect-proof cages. With four whitefly species and three inoculation densities, we had 12 treatments each with 21–37 plants (replicates). After 30 days, the tomato plants in each of the treatments were examined to determine virus infection by PaLCuCNV symptoms and further by PCR.

### Quantification of PaLCuCNV in whiteflies

For quantification of PaLCuCNV DNA in whiteflies after various durations of feeding on PaLCuCNV-infected tomato plants, approximately 600 newly emerged (0–24 h), non-viruliferous adults of each of the four whitefly species were transferred to feed on three PaLCuCNV-infected tomato plants enclosed in three insect-proof cages. Thereafter we randomly collected 10 female adults from leaves of each plant at the end of 0, 24, 48 and 96 h AAP respectively. The insects samples collected were stored at −20 °C and later assayed as sub-samples of 10 female adults each by qPCR for quantifying PaLCuCNV DNA.

### DNA extraction and detection of PaLCuCNV DNA by PCR and qPCR

The DNA from individual whitefly adults was extracted according to the methods of De Barro and Driver (1997)^26^ and Frohlich *et al.* (1999)^27^. Each adult whitefly was placed on parafilm and ground in 30 μl of ice-cold lysis buffer with the round end of a sterile 0.2-ml PCR tube. Lysis buffer was 10 mM Tris pH 8.4, including 50 mM KCl, 0.45% Tween 20, 0.45% Nonidet P-40, 0.2% gelatin and 60 mg/l proteinase K. Extracts were incubated at 65 °C for 1 h and 100 °C for 10 min, and then were centrifuged briefly. The aqueous supernatant was used as template for PCR amplification. All of the DNA samples were confirmed by PCR amplification of an 800 bp fragment of *mtCOI* gene of the whitefly: primer C1-J-2195: 5′-TTGATTTTTTGGTCATCCAGAAGT-3′ and primer L2-N-3014: 5′-TCCAATGCACTAATCTGCCATATTA-3′^27^. Nucleic acids from tomato plants were extracted using the method of Xie *et al.* (2002)^28^. Based on the PaLCuCNV sequence in GenBank (FN256260), the forward primer (5′-TAGTCATTTCCACTCCCGC-3′, corresponding to 312–330 nt) and the reverse primer (5′-TGATTGTCATACTTCGCAGC-3′, corresponding to 938–957 nt) were designed for amplifying a 645 bp segment of PaLCuCNV. 25 μl PCR reactions were carried out containing 2 μl template DNA lysate (concentration not determined), 0.2 μM of each primer, 0.25 mM each deoxynucleoside triphosphates, 1× PCR buffer, and 1U Takara Taq (Takara, Dalian, China). Amplifications were performed in a DNA engine PTC-200 Thermocycler (Bio-Rad, California, USA). Each of the PCR assays contained a negative control of sterile water and a positive control. The annealing temperature was 50 °C for primers of *mtCOI* and 56 °C for primers of PaLCuCNV. The PCR products were electrophoresed on a 1.0% agarose gel in a 1×TAE buffer containing GelRed (Biotium, California, USA) and visualized by a Gel-Doc 2000 system (Bio-Rad, California, USA).

For quantifying PaLCuCNV in the whiteflies, qPCR assays were conducted. For the whiteflies collected in the transmission assay, DNA extraction method was the same as that of conventional PCR assay mentioned above. For quantifying PaLCuCNV DNA in whiteflies following 0, 24, 48 and 96 h AAP, each group was placed on parafilm and ground in 40 μl of ice-cold lysis buffer with the round end of a sterile 0.2-ml PCR tube, and then washed twice using another 100 μl lysis buffer. Extracts were incubated at 65 °C for 1 h and 100 °C for 10 min, and then were centrifuged briefly. The aqueous supernatant was used as template for qPCR amplification. PaLCuCNV *v2* gene was used for quantification with the primers: forward 5′-GACCCGCCGATATAGTCATT-3′, reverse 5′-GTTTGTGACGAGGACAGTGG-3′. *β- actin* gene forward primer 5′-TCTTCCAGCCATCCTTCTTG-3′ and the reverse primer 5′-CGGTGATTTCCTTCTGCATT-3′ were used with each sample as a normalization gene for verifying equal concentrations of whitefly genomic DNA. Amplifications were performed using the SYBR^®^ Premix Ex Taq™ (Takara, Dalian, China) and ABI Prism 7500 fast real-time PCR system (Applied Biosystems, USA) with SYBR green detection (Takara, China) and the relative abundance of viral DNA was calculated using the 2^−Δ Ct^ method.

### Statistical Analyses

A 2 × 4 test of independence was applied to compare frequencies of virus-infected versus uninfected plants between the four whitefly species at each of the three inoculation adult densities. A 2 × 3 test of independence was applied to compare frequencies of virus-infect versus uninfected plants between the three inoculation adult densities for each of the four whitefly species. The relative concentrations of PaLCuCNV in whitefly adults used in the transmission test were compared by a one-way analysis of variance (ANOVA) at a 0.05 significance level followed by LSD tests. The relative virus concentrations as affected by whitefly species (four levels) and AAPs (three levels) were compared by a two-way ANOVA at a 0.05 significance level followed by LSD tests. All the data analyses were performed using IBM Statistics SPSS 20.0.

## Results

### Acquisition of PaLCuCNV DNA by whiteflies

The whiteflies collected after various AAPs were scored for the presence of viral DNA by PCR. PaLCuCNV DNA was detected in 10% of the tested MED adults with a 30 min AAP and in 10% of all the four whitefly species with a 1 h AAP (Table 2). The frequency of detection of PaLCuCNV DNA in the whiteflies increased with the AAP. After a 24 h AAP for MEAM1, MED and Asia II 7, and a 48 h AAP for Asia 1, all individuals acquired PaLCuCNV DNA (Table 2).

### Retention of PaLCuCNV DNA by whiteflies

As detected by PCR, nearly all of the viruliferous adults of each of the four species retained PaLCuCNV DNA for up to 4–5 days after virus acquisition and transfer to feed on cotton plants (Table 3). Thereafter, the proportions of adults with virus DNA in MED, Asia 1 and Asia II 7 declined steadily, down to zero by 25 days in Asia 1 and 50–60% by 40 days in both MED and Asia II 7; in contrast, the proportion of adults with virus DNA detected in MEAM1 individuals remained at 100% until 30 days post virus acquisition and the percentage only dropped to 80% after day 40 (Table 3). PCR tests on the cotton leaves did not detect viral DNA, indicating that the adults had no access to PaLCuCNV when feeding on cotton plants.

### Efficiency of PaLCuCNV transmission by whiteflies

**Figure 1:**
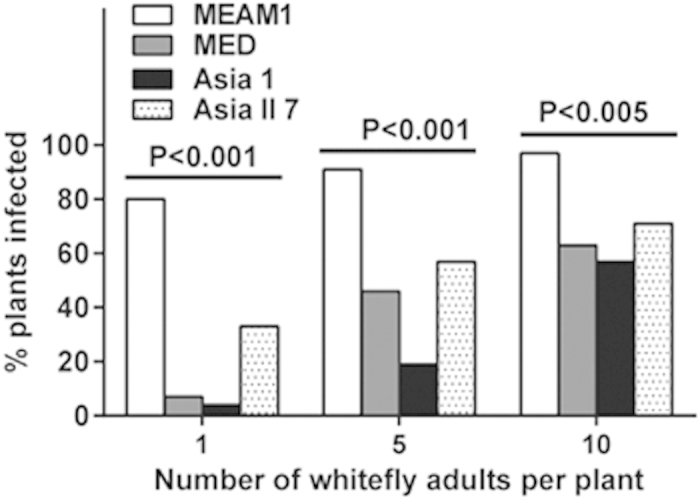
Transmission of PaLCuCNV by four species of the *B. tabaci* complex. Percentages of tomato plants infected by the virus as a function of the number of whiteflies per plant were assessed at 30^th^ day after an inoculation access period of 48 h by viruliferous adults. Frequencies of virus-infected versus uninfected plants between the four whitefly species at each of the three whitefly densities were compared by a 2 × 4 test of independence; for easy comprehension of the comparison between the species, frequency data are presented as percentages in the diagram.

For each of the four whitefly species, some transmission of PaLCuCNV was achieved with a single adult per plant, and the success of transmission increased when the number of adults per plant increased to 5 and 10 (Fig. 1). However, with a single adult per plant the levels of successful transmission were below 10% by both MED and Asia 1, only 33% by Asia II 7, but reached 80% by MEAM1. At each of the three whitefly densities, MEAM1 achieved significantly higher level of transmission than the other three whitefly species (1 adult per plant: χ^2^ = 51.473, df = 3, *P* < 0.001; 5 adults per plant: χ^2^ = 33.197, df = 3, *P* < 0.001; 10 adults per plant: χ^2^ = 13.397, df = 3, *P* = 0.004). In addition, as the number of adults per plant increased from 1 to 10 the levels of successful virus transmission increased significantly in MED (χ^2^ = 19.954, df = 2, *P* < 0.001), Asia 1(χ^2^ = 32.007, df = 2, *P* < 0.001) and Asia II 7 (χ^2^ = 7.700, df = 2, *P* = 0.021). MEAM1 also achieved higher levels of successful transmission as the number of adults per plant increased and approached 100% successful transmission when 10 adults per plant were inoculated; however, the increase was not significant statistically (χ^2^ = 4.257, df = 2, *P* = 0.101) apparently due to the already high level of transmission at 1 adult per plant (Fig. 1).

### The accumulation of PaLCuCNV in whiteflies

**Figure 2:**
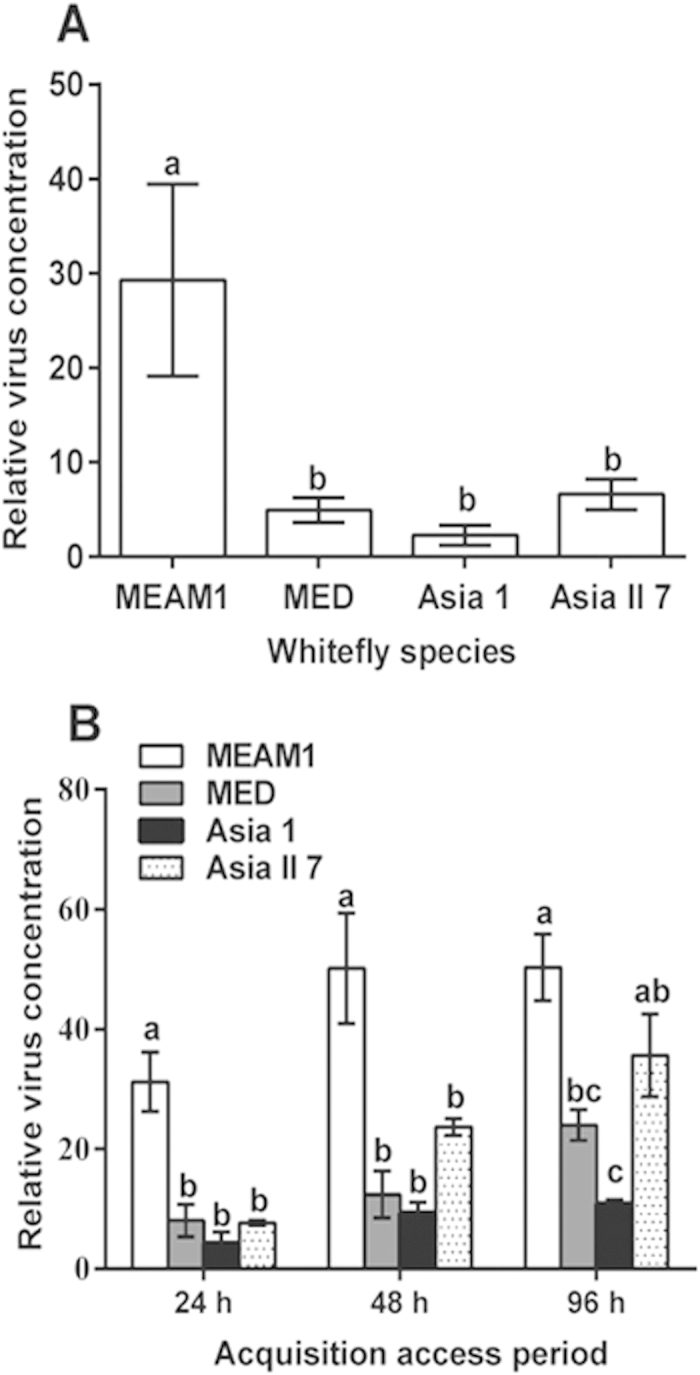
Relative concentration of PaLCuCNV (normalized to the whitefly’s nuclear *β-actin* gene) in four species of the *B. tabaci* complex as quantified by qPCR. (**A**) Following a 48-h acquisition access period, whiteflies were transferred to three true leaf stage tomato plants to feed for a 48-h inoculation access period, and then collected for qPCR tests. Data shown are mean ± SE, n = 15. (**B**) Newly emerged whiteflies were transferred to feed on PaLCuCNV-infected tomato plants for 24, 48 and 96 h and groups of 10 adults were collected for qPCR tests. Data shown are mean ± SE, n = 3. The different lowercase letters above the columns indicate significant differences between whitefly species at P < 0.05 at each of the three acquisition access periods. Relative amount of PaLCuCNV in whiteflies collected at 0 h acquisition access period was zero and not shown here.

Two tests were conducted using qPCR. The first test was to quantify PaLCuCNV in the whitefly adults used in the virus transmission trial. The data showed that the amount of virus in MEAM1 was significantly higher than that in each of the other three species (F_3,56_ = 5.714, P < 0.01; Fig. 2A). The second test was to quantify the virus after three different durations of virus acquisition for each of the four whitefly species. The statistics of the two-way ANOVA indicate that both whitefly species (F_3,24_ = 38.497, P < 0.001) and AAPs (F_2,24_ = 16.490, P < 0.001) had highly significant effects on the relative virus concentrations in the whiteflies (Fig. 2B). However, the interactions of whitefly species×AAPs did not have an significant effect (F_6,24_ = 1.547, P = 0.206). The results show that the relative virus concentration was the highest in MEAM1, followed by Asia II 7 and MED, and finally by Asia 1 (Fig. 2B).

## Discussion

Our data show that all the four cryptic species of the *B. tabaci* complex tested in this study are able to acquire, retain and transmit PaLCuCNV, but the transmission efficiency varied substantially with the whitefly species. MEAM1 transmitted PaLCuCNV to tomato with substantially higher efficiency than did MED, Asia 1 and Asia II 7, while Asia II 7 was a slightly more efficient vector of this virus than MED and Asia 1 (Fig. 1). Although the experiments on virus acquisition, retention and transmission were not formally repeated, these experiments were conducted following preliminary trials. The data of the preliminary trials (Supplementary Table S1–S3) showed a trend of differences between the four species of whiteflies similar to that exhibited by the formal experiments (Fig. 1). In addition, the data indicate that the differences in the ability of PaLCuCNV transmission between the whitefly species were positively associated with the amount of virus in their body (Fig. 2). Altogether, these findings indicate that MEAM1 is a more efficient vector for PaLCuCNV on tomato plants than either MED, Asia 1 or Asia II 7, and this invasive species of whitefly can transmit efficiently begomoviruses that it has not encountered previously.

When offered access to virus-infected tomato plants, it took the whiteflies a minimum of 0–60 min to acquire detectable amount of PaLCuCNV DNA (Table 2). Because whiteflies usually spend some time resting or wandering around after transfer to a new plant^29^ and we did not observe the feeding behaviour of the test whiteflies continuously, thus the exact intervals between the first probes to the successful acquisition of virus DNA were not known, but we may speculate that the minimum AAP was <30 min for MED and <60 min for the other three whitefly species. These records of AAPs are similar to those recorded for MEAM1 and Asia II 3 when offered access to tobacco plants infected by *Tomato yellow leaf curl China virus* (TYLCCNV) or *Tobacco curly shoot virus* (TbCSV)^19^, and to those recorded for MEAM1, MED and Asia II 1 when offered access to tomato plants infected by *Tomato yellow leaf curl virus* (TYLCV)^20^. Some studies demonstrated that MEAM1 could acquire the TYLCV DNA after a 10 min AAP^30^,^31^. In other cases, whiteflies are able to transmit the viruses to new plants with AAPs of 5–60 min^32^,^33^,^34^,^35^. Intervals are observed between the time when virus DNA was detectable in the whitefly body and the time when the whiteflies become capable of infecting new plants^33^, indicating that the virus needs to accumulate to a minimum level and circulate in the whitefly body to make the insect infective. These intervals vary from 6–8 h to >24 h depending on the species/strains of organisms involved^31^,^32^,^34^,^35^.

Following a 48 h AAP on PaLCuCNV infected tomato plants, Asia 1 may retain PaLCuCNV DNA for up to 20 days, while MEAM1, MED and Asia II 7 may retain the virus for 40 days or longer (Table 3). Comparison between the four whitefly species shows clearly that MEAM1 has much higher capability of retaining the virus than the other three species. Variations in capabilities of retaining a given begomovirus has been reported in many cases previously^19^,^20^. Several species of whiteflies have been observed to retain the viral DNA for much longer than the viral capsid protein (CP). For example, although MEAM1 from Israel is unable to transmit the bipartite begomovirus *Abutilon mosaic virus*, following a 4 d AAP on infected abutilon plants both DNA A and DNA B of the virus were detected in the whitefly throughout a period of 15 days of an experiment while the viral CP was only detected in the first seven days^30^,^36^. Rubinstein and Czosnek (1997)^37^ reported that viruliferous MEAM1 could retain TYLCV DNA and transmit the virus for the entire adult life for up to 35 days, although efficiency of viral transmission declined with age. In parallel, these authors were unable to detect the viral CP in the viruliferous MEAM1 after the first 12 days following virus acquisition.

In this study, we demonstrate that MEAM1 can transmit PaLCuCNV more efficiently than the other three species of whiteflies (Fig. 1), and variation in capacity of transmission of the virus may be positively associated with the amount of viral DNA in the whiteflies (Fig. 2). This positive association may indicate that the capability of virus transmission of the whitefly species may be determined, at least partially, by its ability to accumulate the virus in its body. As mentioned in the section of Introduction, begomoviruses are often transmitted by whiteflies in a species/strains specific manner. For example, *Tomato Yellow Leaf Curl China Virus* (TYLCCNV) can be transmitted to tomato and tobacco by MEAM1 and Asia II 3 but not by MED or Asia II 1^19^,^38^. Wei *et al.* (2014)^22^ further demonstrated that secretory cells in the central region of whitefly primary salivary glands play a crucial role in controlling TYLCCNV transmission specificity. Similarly to what has been observed in the current study, Weng *et al.,* (2015)^39^ reported that MEAM1 can transmit *Tomato yellow leaf curl Thailand virus* (TYLCTHV) and *Tomato leaf curl Taiwan virus* (ToLCTWV) more efficiently than the MED, and can transmit TYLCTHV more efficiently than ToLCTWV; Li *et al.*(2010)^20^ report that the invasive MEAM1 and MED can transmit TYLCV more efficiently than the indigenous Asia II 1; Idris *et al.* (2001)^33^ report that the indigenous New World 1 can transmit *Chino del tomate virus* more efficiently than the invasive MEAM1. Brown and Idris (2005)^18^ reported that phylogenetic trees for the *B. tabaci* species complex based on *mtCOI* were in concordance with that of the CPs for the genus *Begomovirus*, suggesting the co-evolutionary histories of begomovirus CPs and their *B. tabaci* vectors. Their analysis suggests that viruses and vectors that originate from the same geographical region are likely to be the most efficient combinations for transmission^18^. For example, field observations in Pakistan provide some circumstantial evidence that the indigenous Asia II 1 plays a more important role in the transmission of cotton leaf curl disease (CLCuD) than the invasive MEAM1^40^,^41^. In contrast, our study indicates that the invasive MEAM1 can transmit PaLCuCNV more efficiently than the two indigenous whitefly species as well as the invasive MED. The physiological and molecular mechanisms underlying these differences warrant future investigations.

The whitefly MEAM1 most likely entered China in the mid to late 1990s, whereas MED was first detected from ornamental plants in Yunnan Province of China in 2003^16^,^42^,^43^. Thereafter, MEAM1 and MED rapidly invaded many regions of China and displaced the indigenous whitefly species in many regions, and MED displaced MEAM1 in some regions^16^,^44^,^45^,^46^,^47^. MEAM1 is still dominant in southern, southeastern and southwestern regions of China’s mainland (Liu *et al.*, unpublished data). Interestingly, from 2007 to 2010 when MEAM1 and MED co-occurred in Henan in the central-north region of China, a pandemic of PaLCuCNV in tomato crops was recorded there^12^. More recently, MEAM1 has been largely displaced by MED in Henan^48^, and only TYLCV is now recorded and PaLCuCNV is no longer recorded from that region (Xue-Ping Zhou, unpublished data). The circumstantial evidence seems to suggest that the recent establishment and then disappearance of PaLCuCNV in Henan is largely determined by the changes of whitefly species in that region.

In conclusion, the current study shows that MEAM1 is an efficient vector of PaLCuCNV and risks of the virus pandemics are high in regions where MEAM1 occurs. Nevertheless, the efficiency of transmission of a virus by a whitefly species may also be affected by the host plants^49^. In China, Li *et al.* (2010)^20^ reported that MEAM1 and MED transmitted TYLCV with similar levels of efficiency, while Ning *et al.* (2015)^50^ reported that MED transmitted TYLCV more efficiently than MEAM1. We noted that these two studies tested the same TYLCV isolate but used different cultivars of tomato in their experiments. Although the whitefly populations of the two species in the two studies were collected from different localities from China and thus the different levels of relative efficiency of TYLCV transmission between the two whitefly species might be partly caused by the differences in whitefly populations, the different cultivars of tomato used in the two studies may also be partly responsible for the relative differences between the two whitefly species observed. Therefore, whether the variation in the ability of PaLCuCNV transmission between the four whitefly species also occurs in other host plants such as papaya and tobacco^15^, are yet to be examined experimentally.

## Additional Information

**How to cite this article**: Guo, T. *et al.* Comparison of transmission of *Papaya leaf curl China virus* among four cryptic species of the whitefly *Bemisia tabaci* complex. *Sci. Rep.* **5**, 15432; doi: 10.1038/srep15432 (2015).

## Supplementary Material

Supplementary Information

## Figures and Tables

**Table 1:** Four species of the whitefly *Bemisia tabaci* complex collected from Zhejiang, Yunnan and Guangdong Province of China and used in the experiments.

**Putative species (biotype names associated)**	**Date of collection**	**Locality of collection**	**Source plant**	***mtCOI*** **GenBank accession no.**
MEAM1 (B)	Sept. 2012	Hangzhou, Zhejiang	Cabbage	KM821540
MED (Q)	June 2009	Ningbo, Zhejiang	Cayenne pepper	GQ371165
Asia 1 (H, M, NA)	Sept. 2012	Honghe, Yunnan	Sweet potato	KC540757
Asia II 7 (Cv)	July 2013	Gaoyao, Guangdong	Hibiscus	KM821541

**Table 2:** Percentage of the four species of whiteflies that acquired PaLCuCNV after various lengths of acquisition access period (AAP) based on detection of viral DNA by PCR. For each of the species and AAPs, 10 adults were assessed.

**Duration of acquisition access period (hours)**	**% adults with PaLCuCNV DNA**			
	MEAM1	MED	Asia 1	Asia II 7
0	0	0	0	0
0.5	0	10	0	0
1	10	10	10	10
1.5	40	10	10	10
2	50	20	20	30
3	70	30	10	40
4	80	70	20	50
6	60	70	40	40
8	80	80	20	40
10	90	80	60	60
12	90	70	90	70
24	100	100	90	100
48	100	100	100	100

**Table 3:** Durations of retention of PaLCuCNV by each of the four species of whiteflies based on detection of viral DNA.

Duration of feeding on cotton (days)*	**% adults with PaLCuCNV DNA**			
	**MEAM1**	**MED**	**Asia 1**	**Asia II 7**
0	100	100	100	100
0.5	100	100	100	100
1	100	100	100	100
2	100	100	100	100
3	100	100	90	100
4	100	100	70	100
5	100	90	100	90
10	100	90	30	90
15	100	90	30	60
20	100	60	30	70
25	100	70	0	60
30	100	60	—†	50
40	80	60	—†	50

^*^Following a 48 h AAP on PaLCuCNV-infected tomato, whiteflies were transferred to feed on cotton for 0 to 40 days. For each whitefly species and duration of feeding on cotton, 10 adults were assessed.

^†^Asia 1 adults were all dead after 30 days of feeding on cotton.
