# Supplementary material for: Comparison of transmission of Papaya leaf curl China virus among four cryptic species of the whitefly Bemisia tabaci complex
Source: Sci Rep. 2015 Oct 21;5:15432. doi: 10.1038/srep15432 (PMC4614018; doi:10.1038/srep15432)
Supplement: Supplementary Information [file srep15432-s1.pdf]

Comparison of transmission of *Papaya leaf curl China virus* among four cryptic species of the whitefly *Bemisia tabaci* complex

Tao Guo[1], Qi Guo[1], Xi-Yun Cui[1], Yin-Quan Liu[1], Jian Hu[2] & Shu-Sheng Liu[1]*
[1]Ministry of Agriculture Key Laboratory of Agricultural Entomology, Institute of Insect Sciences, Zhejiang University, Hangzhou, 310058, China; [2]Yunnan Provincial Key Lab of Agricultural Biotechnology, Biotechnology and Germplasm Resources Institute, Yunnan Academy of Agricultural Sciences, Kunming, 650223, China,

*Correspondence and requests for materials should be addressed to S.S.L. (email: shshliu@zju.edu.cn)

Supplementary Tables 1-3

Supplementary Table S1. Percentage of the four species of whiteflies that acquired PaLCuCNV after various lengths of acquisition access period (AAP) based on detection of viral DNA by PCR. For each of the species and AAPs, 10 adults were assessed.

Duration of acquisition access period (hours)	% adults with PaLCuCNV DNA			
	MEAM1	MED	Asia 1	Asia II 7
0	0	0	0	0
1	20	0	0	10
2	30	20	0	10
3	90	30	10	40
6	80	30	20	80
12	90	60	60	90
24	80	90	50	90
48	100	100	100	100

Supplementary Table S2. Durations of retention of PaLCuCNV by each of the four species of whiteflies based on detection of viral DNA.

Duration of feeding on cotton (days)*	% adults with PaLCuCNV DNA			
	MEAM1	MED	Asia 1	Asia II 7
0	100	100	100	100
1	100	100	100	100
2	100	80	90	100
4	100	90	100	90
5	100	40	100	80
10	100	80	80	90
20	90	30	80	70
30	90	0	0	50

*Following a 48 h AAP on PaLCuCNV-infected tomato, whiteflies were transferred to feed on cotton for 0 to 30 days. For each whitefly species and duration of feeding on cotton, 10 adults were assessed.

† Asia 1 adults were all dead by day 30 of feeding on cotton.

Supplementary Table S3. Transmission of PaLCuCNV by four species of whiteflies after an acquisition access period of 48 h on infected tomato plants and an inoculation access period of 48 h on healthy tomato or tobacco plants as a function of the number of vectors per plant.

Whitefly species	No. of insects per plant	No. of plant tested	No. of plant infected	% of plants infected
MEAM1	1	10	8	80.0
	5	10	9	90.0
	10	10	10	100.0
MED	1	8	1	13.0
	5	8	4	50.0
	10	10	6	60.0
Asia 1	1	5	1	20.0
	5	6	1	17.0
	10	6	3	50.0
Asia II 7	1	10	5	50.0
	5	15	8	53.0
	10	10	8	80.0
